# Phase stability frustration on ultra-nanosized anatase TiO_2_

**DOI:** 10.1038/srep10928

**Published:** 2015-06-04

**Authors:** Snehangshu Patra, Carine Davoisne, Houssny Bouyanfif, Dominique Foix, Frédéric Sauvage

**Affiliations:** 1 Laboratoire de Réactivité et Chimie des Solides, Université de Picardie Jules Verne, CNRS UMR 7314, 33 rue Saint Leu, 80039 Amiens, France; 2 Réseau sur le Stockage Electrochimique de l’Energie (RS2E), FR CNRS 3459, France; 3 Laboratoire de Physique de la Matière Condensée, Université de Picardie Jules Verne, 33 rue Saint Leu, 80039 Amiens, France; 4 IPREM/ECP (CNRS UMR5254), University of Pau, Helioparc, 2 Av. Pierre Angot, 64053 Pau Cedex 9, France

## Abstract

This work sheds light on the exceptional robustness of anatase TiO_2_ when it is downsized to an extreme value of 4 nm. Since at this size the surface contribution to the volume becomes predominant, it turns out that the material becomes significantly resistant against particles coarsening with temperature, entailing a significant delay in the anatase to rutile phase transition, prolonging up to 1000 °C in air. A noticeable alteration of the phase stability diagram with lithium insertion is also experienced. Lithium insertion in such nanocrystalline anatase TiO_2_ converts into a complete solid solution until almost Li_1_TiO_2_, a composition at which the tetragonal to orthorhombic transition takes place without the formation of the emblematic and unwished rock salt Li_1_TiO_2_ phase. Consequently, excellent reversibility in the electrochemical process is experienced in the whole portion of lithium content.

Going towards the nano-sizing of functional materials led to the discovery of new properties and new reactivities triggered by the exacerbation of the surface properties with respect to the generally better known and more controlled bulk properties^1^. Since the specific example of the graphene and carbon nanotubes back to early 1990’s^2^, the research on materials nanostructuration has shown tremendous accomplishments, not only for the carbon, but it was also extended to all kinds of sulfides^3^ and oxides^4^,^5^ materials. This has contributed to the emergence of the nanoionics research area raising significant fundamental and practical expectations. It has fostered important breakthroughs in energy-related applications, as in photovoltaics including dye-sensitized solar cells^6^ or quantum-size absorbers for multi-exciton generation and tunable absorption energy^7^,^8^, in supercapacitors^9^,^10^ and in lithium-ion batteries (LiB)^11^,^12^,^13^. The nanostructuration of electrode materials has opened up the utilization of poor electronic/ionic mixed conductors and was also shown to noticeably enhance the power rate capability of the electrode^14^. The successful research maturation of carbon-coated nanocrystalline olivine LiFePO_4_^15^ is one of the best examples despite its electronic conductivity of 10^−9^ S/cm and even lower lithium-ion conductivity^16^.

TiO_2_ is undeniably the inorganic semi-conductor which is the most studied worldwide. It is the leading contender material for photocatalysis^17^, introduced in biomedical implants^18^,^19^ or in more traditional applications such as white pigment, metallurgy etc. It exhibits an exceptionally rich polymorphism by gathering 11 different types of TiO_2_ structures, among which 6 are stable under ambient conditions and 5 are high pressure phases. The thermodynamically most stable structure under ambient conditions is the rutile followed by the brookite and the anatase^20^,^21^. Interestingly, downsizing TiO_2_ towards nano has led to surprising discoveries. One of those regards the size dependency of the phase stability diagram. This was highlighted by Banfield *et al.* who concluded a size crossover threshold of 14 nm below which the anatase TiO_2_ becomes in reality the most stable polymorph^22^. This size dependence was also confirmed by Navrotsky *et al.* using calorimetric solid/air surface energy measurements and solution calorimetry, concluding a crossover when the BET surface area becomes greater than 50 m^2^.g^−1^, corresponding to a size of 30 nm assuming spherical particles. This crossover in the relative polymorphism stability stems from the five times lower surface enthalpy of the anatase which modifies significantly the total free Gibbs energy of the system when reducing the size^23^,^24^. A second example concerned the modification of the electrochemical characteristics of the anatase TiO_2_ when going towards the nano^25^. The lithium insertion in bulk anatase TiO_2_ basically undergoes one first order phase transition at *ca.* 1.75 V (vs. Li^+^/Li) involving the coexistence in the grain of an anatase lithium-poor Li_α_TiO_2_ phase (S.G. I41/amd; α ≈ 0.01) and a lithium-rich orthorhombic phase Li_β_TiO_2_ (S.G. Imma; 0.5 < β < 0.6)^26^,^27^,^28^. The complete filling of lithium cation into the octahedral interstitial site, leading to the end member Li_1_TiO_2_ composition, can be reached solely if the particle size is sufficiently reduced. It occurs below 1.5 V through a second biphasic reaction separating the orthorhombic Li_β_TiO_2_ and Li_1_TiO_2_ for which the structure corresponds to a tetragonal lattice cell similar to the anatase with *ca.* 3.5% lattice volume expansion^26^,^28^. Reaching this completely filled composition is basically circumvented since it penalizes the electrode coulombic efficiency as a result from an important lack of lithium transport throughout the crystal structure^29^. Wagemaker *et al.*^30^ highlighted a phase segregation between Li_α_TiO_2_ on the one hand and Li_β_TiO_2_ on the other hand when the particles are ranging in the nanoscale domain. This phenomenon is driven by a thermodynamic effect resulting from a more favorable minimization of the free enthalpy of the system when the phases are separating rather than the coexistence of two phases in one grain. A similar process has been revealed in other nanocrystalline biphasic materials such as LiFePO_4_/FePO_4_ and Li_4_Ti_5_O_12_/Li_7_Ti_5_O_12_^31^,^32^. Lastly, the reduction of miscibility gap when decreasing the particle size was also exposed. This translates into the extension of the α solid solution domains from 0.01 to 0.21 Li^+^ when going from 140 to 7 nm TiO_2_^30^; a feature also experienced in the case of the hematite α-Fe_2_O_3_^33^ and the olivine LiFePO_4_^34^ even though the defect chemistry associated to the reduction of size may also contribute to this phenomenon.

In the present study, we reveal the exceptional robustness of the anatase crystal structure when an extremely small particle size, of *ca.* 4 nm, is reached (S_BET_ ≈ 300 m^2^.g^−1^). This translates into an abnormally high temperature for anatase to rutile transformation. We are also exposing a complete vanishing of the miscibility gap upon lithium insertion which transforms the galvanostatic curve from a classic potential/composition plateau into a S-shape profile along the full range of lithium composition, without ending up to the formation of the rock-salt LiTiO_2_. The relevance of this new feature on the electrochemical performances of the particles and the fundamental and practical outcomes of such findings is herein discussed.

## Results and discussion

**Figure 1:**
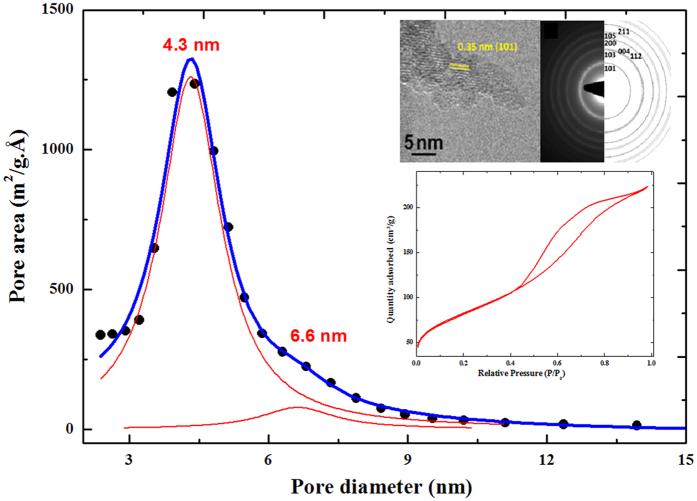
Distribution of the pore area as a function of the pore diameter using the B.J.H. method for the 4 nm size anatase TiO_2_. In inset is reported the high resolution transmission electron microscopy (HRTEM) micrograph and the corresponding selected area electron diffraction pattern (SAED) of the particles and the adsorption/desorption nitrogen isotherm.

Anatase and brookite irreversibly transform into the denser rutile phase at around 650–700 °C under air^35^. In this study, we are comparing two size of particles, 4 nm synthesized by a two steps room-temperature process^36^ and 20 nm particles synthesized by a hydrothermal synthetic route originally adapted for dye-sensitized solar cells (*ca.* 80 m^2^.g^−1^)^37^. The size of the particles have been determined by three main techniques: direct observation by transmission electron microscopy (TEM), x-ray analysis using Williamson-Hall formalism and Raman spectroscopy using the phonon confinement model^37^,^38^. A particular attention has been paid in this work to obtain relatively well mono-disperse particle size in order to avoid inhomogeneous behavior between larger and smaller particles upon heating or during electrochemical measurements. The shape of the nitrogen adsorption/desorption isotherm features a typical type-IV isotherm characteristic of a mesoporous material (Fig. 1)^39^. The hysteresis loop is assimilated to an A-type using DeBoer classification suggesting an open mesoporosity for which the size is invariant along the pore length^40^. We determined a B.E.T. surface area of 288 m^2^.g^−1^ (±1 m^2^.g^−1^) by analysis of the nitrogen adsorption part using a 6 points method (C_BET_ = 80). Analysis of the desorption part using the Barrett-Joyner-Halenda (B.J.H.) method is reported in Fig. 1. The pore distribution features a bimodal distribution with a main contribution at 4.3 nm and a second minor one at 6.6 nm. The morphology of the particles are assimilated to needles made of well-crystallized and well-monodisperse nanoparticles of 4-5 nm all aligned along the (101) plane (d_hkl_ = 0.35 nm). This direction corresponds to the low energy facet of the anatase TiO_2_^41^,^42^. This peculiar morphology results from a solid-state self-reorganization of the nanoparticles during the crystallization process^37^. XPS experiments were carried out to characterize the chemical properties of the 4-5 nm size particles which are synthesized in diluted 0.1 M NH_4_F_(aq)_ medium (pH ≈ 6). The XPS spectrum of the Ti 2p core peak is showing, as expected for TiO_2,_ a strict Ti^4+^ valence state profile with the 2p^3/2^ and 2p^1/2^ components at 458.9 eV and 464.6 eV, respectively (Fig. S1). This is in agreement with a O1s peak located at 530.1 eV and a O/Ti ratio = 2.1. Fluoride traces are found at the surface of sample to an extent of only 0.6% (atomic %). The binding energy of F 1s is 684.2 eV consisting of adsorbed fluoride instead of Ti-F bonds at the surface of the crystal structure^43^,^44^.

**Figure 2:**
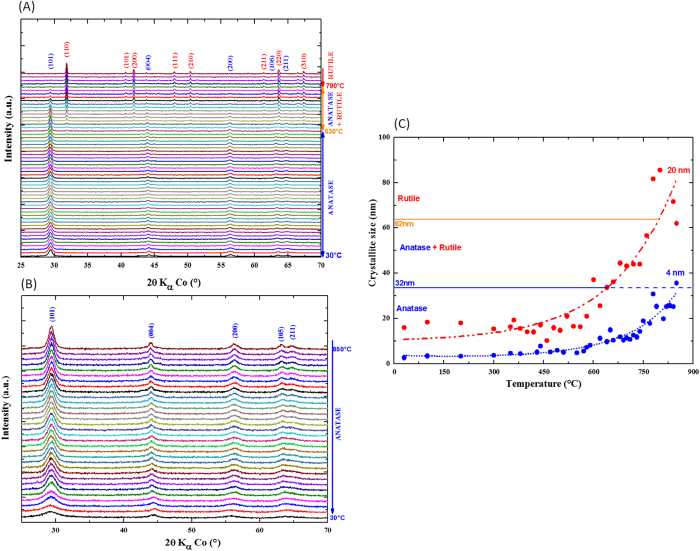
*In situ* x-ray diffraction study with temperature for (**a**) 20 nm-based anatase TiO_2_ (**b**) 4 nm-based anatase TiO_2_. **(c)** Evolution of the crystallite size as a function of temperature derived from the *in situ* x-ray experiments.

*In situ* x-ray diffraction was used to monitor the evolution of the structure and the microstructure as a function of temperature, comparing the 20 nm-based particles (Fig. 2a) with the 4 nm counterpart (Fig. 2b). For the first size, the evolution of the diffractogram as a function of temperature highlights the onset at *ca.* 630 °C of new diffraction peaks at 2θ = 31.82, 40.76, 41.84, 47.94, 50.37°(…) ascribed to the (110), (101), (200), (111), (210) (…) reflections of the rutile polymorph. The rutile grows at the expense of the anatase structure. The two phases are coexisting between 630 °C to 790 °C before turning completely into rutile under our experimental conditions. By comparison, the thermal stability of the 4 nm is drastically changed. No phase transition is noticed even at temperatures of as high as 850 °C. Note that this temperature threshold corresponds to the upper limit of the furnace used for this *in situ* x-ray diffraction study. Whereas the experimental procedure was rigorously the same, such a difference clearly highlights the main implication of the particle size in the thermal stability of the anatase. Nevertheless, a slight contribution of the low amount adsorbed fluoride upon the anatase TiO_2_ surface cannot be rule out since this latter is reported to also stabilize the energy surface of the anatase; consequently to contribute in the delaying of the anatase-to-rutile transition temperature to a range of 800 °C to 900 °C in air^45^,^46^.

A very similar experiment was performed by *in situ* Raman spectroscopy showing that in fact anatase to rutile transition takes place at a temperature of as high as 1000 °C in the 4 nm particles (Fig. S2). Rutile was observed in the same range of temperatures as x-ray diffraction for the particles of 20 nm. This is the first time, to our knowledge, that such a so high thermal durability of the anatase TiO_2_ is demonstrated. This extended thermal stability at the nanoscale can find interest for some applications such as (photo)-catalysis for instance in which the anatase polymorph is well-established to exhibit higher catalytic activity than the rutile polymorph^47^.

The evolution of crystallite size as a function of temperature for the two samples was calculated by using Williamson-Hall formalism. The evolution is gathered in Fig. 2c. It features an exponential increase of crystallite size as a function of temperature in which the onset depends on the size of the particles, *ca.* 550 °C for the 20 nm particles versus *ca.* 700 °C for the smaller particles. Interestingly the shape of the exponential also differs; the rise being steeper in the case of the bigger particles. The greater robustness of anatase crystal structure against temperature is ascribed to the deferred process of particles coalescence and grain coarsening. This comes in good agreement with the literature which has hypothesized grain coarsening to be the main factor directing the transition to rutile as a result from the stepwise decrease of surface energy contribution to the total free Gibbs energy^48^. The delayed coarsening of the 4 nm nanoparticles is a direct consequence of the greater surface stability. The reduction of particle size from 20 to 4 nm has an important impact on the surface to volume ratio. This latter increases from 0.3 to 1.5 if we assume the particles to have spherical morphology. In other words, this ratio means that for the smallest particles, the surface contribution to the volume predominates by contrast to the 20 nm size particles for which the bulk properties are still prevailing.

Based on this *in situ* XRD experiment, we evaluated that the transition towards rutile starts when the crystallite size attains a value of around 32 nm. Interestingly, such value is never reached regardless of the temperature in the case of the 4 nm particles. Anatase is completely transformed to rutile when the sizes of the crystallites reach around 60 nm. These values obtained by *in situ* x-ray method also corroborate relatively well the set of numbers reported by Banfield *et al.* based on another approach relying on the expression of the surface energies from monitoring isothermal coarsening kinetics of nano-sized TiO_2_^48^.

**Figure 3:**
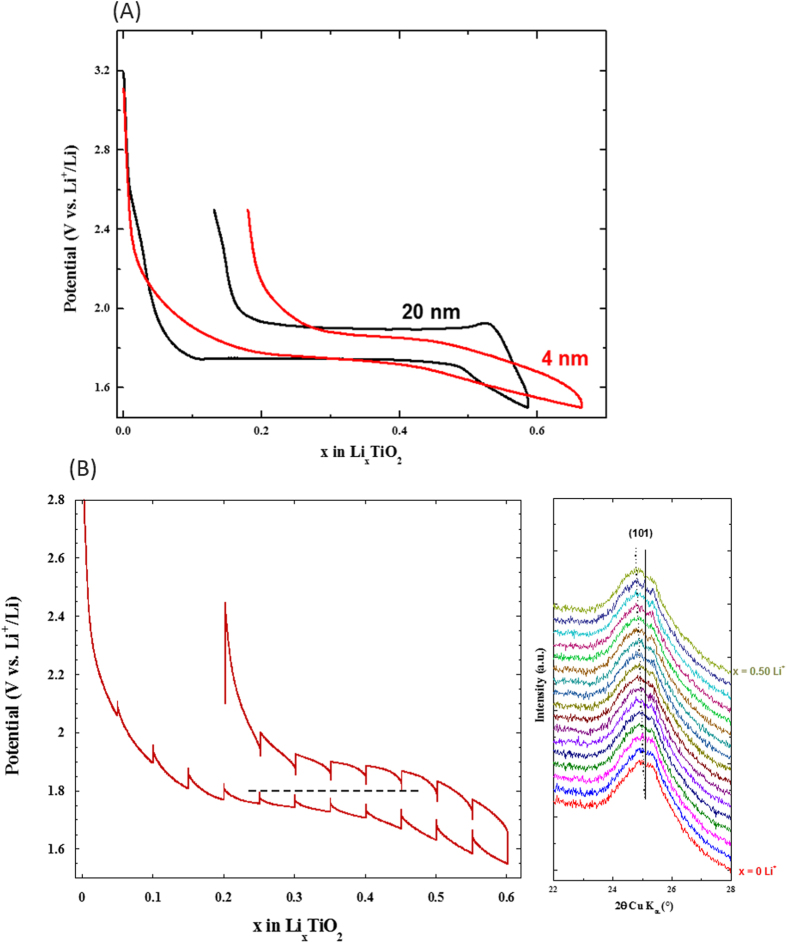
(a) Comparison of the galvanostatic lithium insertion/de-insertion curve between anatase TiO_2_ of 20 nm size and 4 nm size (C/20 rate corresponding to the insertion of one lithium every 20 hours).(**b**) Galvanostatic Intermittent Titration Technique (GITT) curve of the 4 nm-size anatase TiO_2_ (C/20 rate and 50 hours relaxation time) and the corresponding *in situ*/*in operando* x-ray diffraction study.

The exceptional stability of the anatase crystal structure at extreme nano size is also verified by electrochemical means in a remarkable manner. In this case, the interface becomes different compared to the previous section. Previously, the high robustness of the anatase crystal structure was derived from the low solid/gas surface energy. In the case of a conventional lithium-ion battery configuration, the interfaces become more complex. The predominant one corresponds to a solid/liquid (electrolyte) interface, TiO_2_/TiO_2_ contacts remain theoretically unchanged and a new solid/solid interface appears between TiO_2_ and the conducting carbon (KetjenBlack 600). Also note that no binder was used in the electrode formulation to avoid any additional components. Figure 3a compares the first cycle of galvanostatic discharge/charge of the electrode between anatase TiO_2_ of 20 nm and the 4 nm using an intermediate cycling rate of C/20 (*ie.* one lithium inserted/de-inserted every 20 hours). For the preparation of the composite electrodes, the active material is manually mixed with Ketjen Black 600-type carbon (denoted as KB600) in a mortar (see experimental section). It results in the destruction of the original needles leading to individual spherical nanoparticles well dispersed within the electron percolating carbon network. The lithium insertion/deinsertion process into/from the 20 nm particles features a distinct potential-composition plateau at *ca.* 1.75 V (vs. Li^+^/Li) involving the insertion of 0.58 lithium cation at a cutoff potential of 1.5 V (vs. Li^+^/Li). This plateau corresponds to the well-established bi-phasic reaction separating the quadratic lithium-poor Li_α_TiO_2_ phase (S.G. I41/amd) and the orthorhombic lithium-rich Li_β_TiO_2_ phase (S.G. Imma). The values of α and β are a function of the particle size^30^. For the size of 20 nm, we found values for α and β equal to *ca.* 0.11 and *ca.* 0.50, respectively. However, when decreasing further the size to 4 nm, there is a drastic modification in the shape and capacity of the galvanostatic discharge/charge curve which is then characterized by a monotonous potential decrease/increase of potential during lithium insertion/deinsertion process. This S-shape galvanostatic curve is typically credited to a solid solution in which the lithium is inserted without long range ordering into the octahedral interstitial sites. Last but not least, this solid solution-like curve also comes with an enhancement in the electrochemical capacity of the material since this latter can host 0.67 Li^+^ per formula unit at a cutoff potential of 1.5 V (vs. Li^+^/Li), compared to 0.58 Li^+^ for the 20 nm sample. Such a difference translates into a gain in gravimetric capacity of around 30 mAh.g^−1^. ICP-AES determination of the lithium content on the smallest size TiO_2_ was giving 0.65 lithium-ion per formula unit in excellent agreement with the Coulomb law.

**Figure 4:**
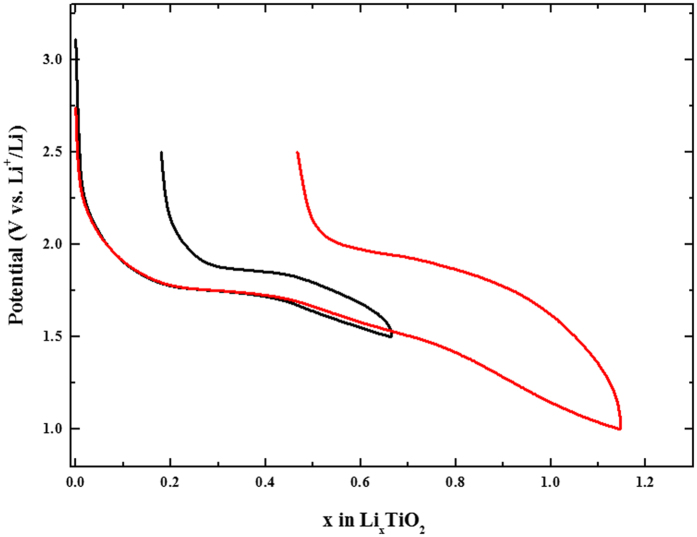
Comparison of the first cycle galvanostatic lithium insertion/de-insertion curve of the 4 nm size particles (C/20 rate) between 1.5 V and 1.0 V (vs. Li^+^/Li) cutoff potential.

The demonstration of the complete solid solution-type insertion at nanoscale is verified by electrochemical means, *in situ*/*in operando* x-ray diffraction study and by selected area electron diffraction study. The galvanostatic intermittent titration technique (G.I.T.T.) reported in Fig. 3b highlights first the low polarization of the material lying between 35 to 70 mV despite no electrode formulation and optimization was carried out (C/20 discharge rate and 50 hours relaxation time). This originates from the shortened electronic and ionic motion path in the solid. The evolution of potential at equilibrium condition confirms the absence of a hindered voltage-composition plateau in the whole portion of composition between α and β. This G.I.T.T. was combined to *in situ* x-ray diffraction study between TiO_2_ and Li_0.5_TiO_2_ even though the diffraction peaks are relatively broad owing to the nanosize character of the particles. The results are self-consistent with a complete solid solution domain as witnessed by the continuous shift of the (101) diffraction plane towards lower diffraction angles during lithium insertion and the absence of the orthorhombic Li_β_TiO_2_ phase appearing at the expense of the quadratic Li_α_TiO_2_ (Fig. 3b). Such a vanishing of the miscibility gap when the particles size becomes as low as 4 nm impeccably completes the phase stability diagram reported by Wagemaker *et al.* based on neutron diffraction study for particles lying between 120 to 7 nm^30^. For this last size, the authors determined values of α and β equal to 0.21 and 0.67, respectively. They also marked out the extent of the kinetically restricted γ phase, corresponding to the quadratic Li_1_TiO_2_ composition, to rise exponentially when the size of the particles decreases. Along this line, Sudant’s *et al.* achieved to reach electrochemically the Li_1_TiO_2_ composition as a single phase at a size of 6-7 nm, even though the typical electrochemical plateau of the two-phase reaction is not clearly noticed^49^. A well-defined plateau separating β and γ phases was only noticed on micrometric materials combined to polymer electrolytes at higher temperatures than ambient^50^,^51^. The fully discharged composition can be obtained under room temperature conditions at lower potential than 1.5 V (Fig. 4). For cut-off potential of 1 V, the material can insert 1.15 Li^+^ corresponding to a first discharge capacity of approximately 385 mAh.g^−1^ (Li_1.15_TiO_2_). This capacity is slightly greater than the theoretical capacity of TiO_2_ (337 mAh.g^−1^) and the experimental capacities on anatase nanorods (Q = 320 mAh.g^−1^, Li_0.95_TiO_2_)^52^ and the 6 nm particles of Sudant *et al.*^49^. However, it should be noted that this capacity enhancement is also coming with an increase of the first cycle irreversibility from 0.18 to 0.46 Li^+^ (Fig. 4). This extra-capacity does not originate from additional lithium storage ability since the crystal structure cannot host more than 1 lithium cation per formula unit. EELS spectroscopy on this discharged sample does not reveal any existence of Ti^2+^ which could have been attributed for instance to an anionic redox process at low cell voltage, *ie.* electrochemical formation of TiO_2-δ_. TEM investigation at different states of discharge (x = 0, 0.5, 0.9, 1.1 and 1.5 Li^+^) suggests this extra-capacity to arise in part or in whole from an early electrolyte degradation at low potential leading to the formation of a solid electrolyte interphase (SEI) in good agreement with recent observations made on iron-doped TiO_2_ nanoparticles synthesized by a very close procedure (Fig. SI3)^53^.

**Figure 5:**
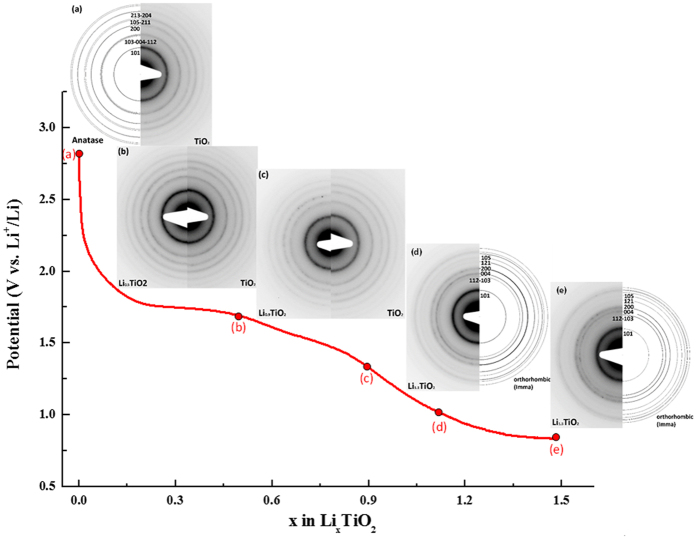
Evolution of the SAED pattern during first discharge of the 4-nm Li_x_TiO_2_ system.

**Figure 6:**
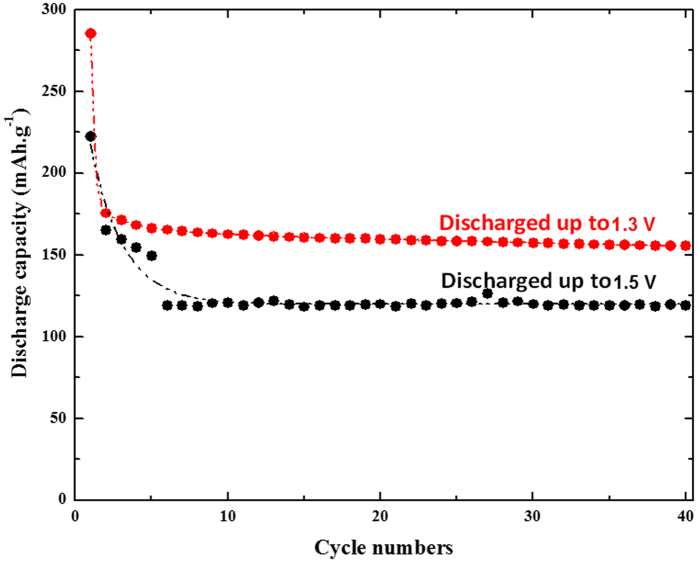
Gravimetric capacity retention curve at C/20 rate for the 4 nm-based anatase TiO_2_ discharged either to 1.5 V (vs. Li^+^/Li) or to 1.3 V (vs. Li^+^/Li).

The selected area electron diffraction patterns for the different lithiated samples provide more local information on the lithiation procedure at nanoscale. The results, gathered in Fig. 5, are first confirming the complete solid solution domain until it reaches a composition very close to Li_1_TiO_2_. Indeed, for x = 0.9 Li^+^, the resulting SAED pattern is still indexed with the anatase crystal structure by contrast to the composition of x = 1.1 Li^+^ for which the diffraction pattern does not match anymore. Along this remarkable extension of the solid solution domain, only the positions of the rings are modified as a result from lattice cell volume evolution during the lithium ion intake. The refinement of the ring position for the different crystal orientations shows upon insertion that both a and c parameters are increasing (Table 1). A distinctive feature comes from the very surprising cell volume expansion along this solid solution which reaches a maximum of 21% between the anatase TiO_2_ (V_cell_ = 132.0 Å^3^) and the anatase Li_0.9_TiO_2_ (V_cell_ = 159.7 Å^3^). Such an uncommon value of cell expansion without evident particles fracturing denotes the excellent flexibility of the anatase framework at the nanometric scale. For lithium composition beyond 0.9 Li^+^, the quadratic symmetry is broken and the SAED patterns can be indexed solely with the orthorhombic Li_β_TiO_2_ phase (S.G. Imma). This phase transition is likely driven by stress relaxation since the cell volume shrinks considerably to a more common value of 140.7 Å^3^. The β value in the literature ranges between 0.5 to 0.7 Li^+^ 30. In this work we demonstrate that in practice β can attain the maximum filling of the octahedral sites by lithium. Consequently, the quadratic rock-salt structure Li_1_TiO_2_ solved first by Cava *et al.* becomes less stable at nanoscale than the orthorhombic counterpart^26^. We explain the modification of the electrochemical phase diagram to stem at once from the enhancement of the structure flexibility of the anatase at nanoscale and the dominance of the energy surface on the total free Gibbs energy. Interestingly, bypassing the rock-salt structure has numerous implications. The first one concerns the excellent capacity retention in all portions of the solid solution domain without any electrode formulation optimization (Fig. 6). Typically the formation of the rock-salt Li_1_TiO_2_ was always avoided in practice owing to its poor electrical properties. Therefore lithium insertion into the anatase TiO_2_ was always restricted to the formation of Li_β_TiO_2_. Because of the miscibility gap loss, we managed to cycle in the full range of lithium composition. This corresponds to a noticeable gain in capacity of *ca.* 65 mAh.g^−1^ and almost 130 mAh.g^−1^ in comparison to a conventional anatase TiO_2_ with a value of β of around 0.5 Li^+^. Nevertheless, although the capacity at potentials lower than 1 V is increasing with the particle decrease, likely due to SEI formation, the elimination of the rocksalt Li_1_TiO_2_ structure at nanosize does not pave the way towards the possibility of proceeding in the lithium conversion reaction of the anatase TiO_2_ yielding to Ti^0^ embedded in Li_2_O-based matrix.

## Conclusions

In this work, we established the high robustness of the anatase crystal structure when going to extreme nano-sizing as a result from the very low energy surface of the anatase which, when the surface prevails to the total volume, affects the established phase stability diagram whether it is solid/air interface and solid/electrolyte interface in a lithium-ion battery. This translates into a delayed process of grain coarsening causing a significantly higher temperature required to onset the anatase to rutile transition. The mechanism of lithium insertion is also strongly modified. The two characteristically reported two-phase insertion processes yielding to the quadratic rock-salt Li_1_TiO_2_ is transformed at extreme nanosize into a complete solid solution domain until almost the full lithium occupation into the anatase. The kinetically restricted rock-salt structure is no longer stable at nanosize, at which it is replaced by an orthorhombic Li_1_TiO_2_ similar to the previously reported Li_0.5_TiO_2_. As a result, an excellent reversibility in the electrochemical processes can be obtained without any electrode formulation optimization in the whole portion of the solid solution domain (until x = 0.9 Li^+^). Nonetheless the development of an optimized electrode formulation will be required to fully take advantage of this complete solution domain with the aim to fully exploit the theoretical capacity of the anatase TiO_2_.

## Methods

The synthesis of the 4 nm particles was performed as follows: 30 mL Ti(iOPr)_4_ (titanium (IV) isopropoxide) was added to 300 mL of water under vigorous stirring for 4 hours to ensure complete hydrolysis. The white precipitate was then retrieved by vacuum filtration or centrifugation, washed several times with water followed by ethanol and dried at room temperature overnight. It was then divided into several batches and kept for ageing from several days to 3 months at room temperature (20 °C) in a solution of NH_4_F_(aq)_ of 0.1 mol.L^−1^ 36. The 20 nm particles were synthesized by a hydrothermal route. To do so, 0.1 mol.L^−1^ of titanium isopropoxide (29.3 g) was mixed with an equimolar proportion of acetic acid (6 g) under stirring. The solution was kept under vigorous stirring for 15 min and transferred into 175 mL water for titanium hydrolysis. The solution was kept for 1 hour. 1 mL of nitric acid is then added and the solution peptized at 78 °C for 90 minutes (ramp of 45 min). The solution turns from white to light blue. The solution was then concentrated with a rotavap (50 °C/60 mBar) until 60 g is obtained. The resulting solution is autoclaved at 250 °C for 12 hours in a Parr Teflon vessel (Volume 100 mL)^54^.

Titanium isopropoxide and ammonium fluoride were purchased from Aldrich and used as received. Transmission Electron Microscopy (TEM) and Selected Area Electron Diffraction were performed using a high resolution FEG FEI Tecnai F20 S-TWIN with accelerating tension of 200 kV. The simulations of SAED patterns were carried out using Electron Diffraction Software. Lithiated TiO_2_ samples were retrieved from the battery in an Ar-filled glove box, carefully washed with DMC and recovered by centrifugation inside the glove box. The sample was transferred into the microscope column without any air exposure.

*In situ* x-ray diffraction patterns were acquired using a Be-based electrochemical cell suited to a Bruker D8 diffractometer (CuK_α_, λ = 1.54056 Å). *In situ* x-ray diffraction with temperature was performed on a Bruker D8 diffractometer with a Co radiation (λ_1_ = 1.78897 Å, λ_2_ = 1.79285 Å) equipped with an Anton Parr Chamber HTK from room temperature to 850 °C. Each pattern was recorded in air with a heating rate of 0.08 °C.s^−1^. Raman measurements were performed using the 514.5 nm line from an argon ion laser and analyzed using a Jobin Yvon T64000 spectrometer equipped with a charge coupled device. An optical microscope was used to focus the incident light as a spot of about 2 μm in diameter on the sample. The temperature dependence of the Raman spectra was measured using a TS1500 Linkam stage from room-temperature to 1100 °C with a heating rate of 10 °C.min^−1^. XPS measurements were carried out with a Kratos Axis Ultra spectrometer, using a focused monochromatized Al Kα radiation (hυ = 1486.6 eV). The XPS spectrometer was directly connected through a transfer chamber to an argon dry box, in order to avoid moisture/air exposure of the samples. For the Ag 3d^5/2^ line, the full width at half maximum (FWHM) was 0.58 eV under the recording conditions. The analysed area of the samples was 300 × 700 μm^2^. Peaks were recorded with a constant pass energy of 20 eV, and 160 eV for the survey spectra. The pressure in the analysis chamber was around 5.10^−9^ mbar. Short acquisition time spectra were recorded before and after each normal experiment to check that the samples did not suffer from degradation during the measurements. The binding energy scale was calibrated from the hydrocarbon contamination using the C1s peak at 285.0 eV. Core peaks were analysed using a nonlinear Shirley-type background^55^. The peak positions and areas were optimized by a weighted least-squared fitting method using 70% Gaussian, 30% Lorentzian lineshapes. Quantification was performed on the basis of Scofield’s relative sensitivity factors^56^.

All electrochemical tests were performed in two-electrode Swagelok-type cells. For this, the active material was mixed in a mortar with 15% in mass of KB600. The electrochemical cell consisted of around 10 mg of active material separated from a lithium foil by two fiberglass sheets soaked by a LP30 Merck electrolyte (1M LiPF_6_ in 1:1 EC/DMC). Galvanostatic experiments were performed using a VMP3 galvanostat/potentiostat. For Galvanostatic Intermittent Titration Technique experiments (GITT), the cells were intermittently discharged/charged for 30 minutes at C/20 rate with open circuit voltage periods of 50 hours.

## Additional Information

**How to cite this article**: Patra, S. *et al.* Phase stability frustration on ultra-nanosized anatase TiO_2_. *Sci. Rep.* **5**, 10928; doi: 10.1038/srep10928 (2015).

## Supplementary Material

Supplementary Information

## Figures and Tables

**Table 1:** Evolution of lattice cell parameter determined by SAED as a function of lithium composition in TiO_2_.

	**TiO_2_**	**Li_0.5_TiO_2_**	**Li_0.7_TiO_2_**	**Li_0.9_TiO_2_**	**Li_1.1_TiO_2_**	**Li_1.5_TiO_2_**
a (Å)	3.78	3.89	3.94	4.10	3.98	4.00
b (Å)	3.78	3.89	3.94	4.10	3.98 (≠ a)	4.00 (≠ a)
c (Å)	9.24	9.16	9.28	9.5	8.88	8.72
V (Å^3^)	132.0	138.6	144.1	159.7	140.7	139.5
Type	Anatase I41/amd	Anatase I41/amd	Anatase I41/amd	Anatase I41/amd	Li_0.5_TiO_2_ Imma	Li_0.5_TiO_2_ Imma
