# Supplementary material for: Phase stability frustration on ultra-nanosized anatase TiO2
Source: Sci Rep. 2015 Jun 4;5:10928. doi: 10.1038/srep10928 (PMC4455250; doi:10.1038/srep10928)

Phase stability frustration on ultra-nanosized anatase TiO_2

Snehangshu Patra,[1,2] Carine Davoisne,[1,2] Houssny Bouyanfif,[3] Dominique Foix,[2,4] Frédéric Sauvage[1,2]*

[1]Laboratoire de Réactivité et Chimie des Solides, Université de Picardie Jules Verne, CNRS UMR 7314, 33 rue Saint Leu, 80039 Amiens, France

[2]Réseau sur le Stockage Electrochimique de l'Energie (RS2E), FR CNRS 3459, France

[3]Laboratoire de Physique de la Matière Condensée, Université de Picardie Jules Verne, 33 rue Saint Leu, 80039 Amiens, France

[4]IPREM/ECP (CNRS UMR5254), University of Pau, Helioparc, 2 Av. Pierre Angot, 64053 Pau Cedex 9, France

Figure SI-1: Low resolution XPS spectrum and high resolution XPS spectrum at Ti 2p level of the 4 nm TiO_2 particles synthesized at room-temperature in 0.1M NH_4F solution.

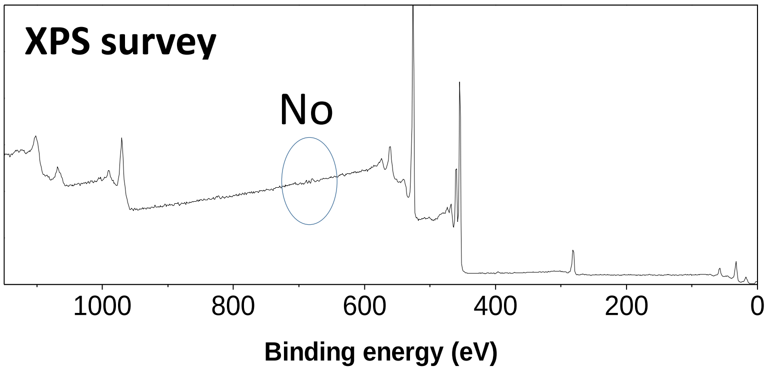

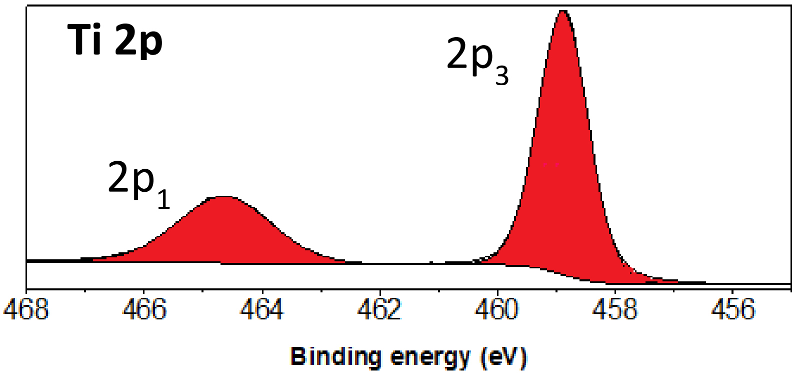

Phase stability frustration on ultra-nanosized anatase TiO_2

Snehangshu Patra,[1,2] Carine Davoisne,[1,2] Houssny Bouyanfif,[3] Dominique Foix,[2,4] Frédéric Sauvage[1,2]*

[1]Laboratoire de Réactivité et Chimie des Solides, Université de Picardie Jules Verne, CNRS UMR 7314, 33 rue Saint Leu, 80039 Amiens, France

[2]Réseau sur le Stockage Electrochimique de l'Energie (RS2E), FR CNRS 3459, France

[3]Laboratoire de Physique de la Matière Condensée, Université de Picardie Jules Verne, 33 rue Saint Leu, 80039 Amiens, France

[4]IPREM/ECP (CNRS UMR5254), University of Pau, Helioparc, 2 Av. Pierre Angot, 64053 Pau Cedex 9, France

Figure SI-2: *In situ* Raman spectroscopy with temperature on 4 nm anatase TiO_2. Rutile Raman spectra are observed for temperatures above 1000°C. Note the disappearance above 1000°C of the strong band at about 150cm^{-1} characteristic of the Anatase to Rutile phase transition

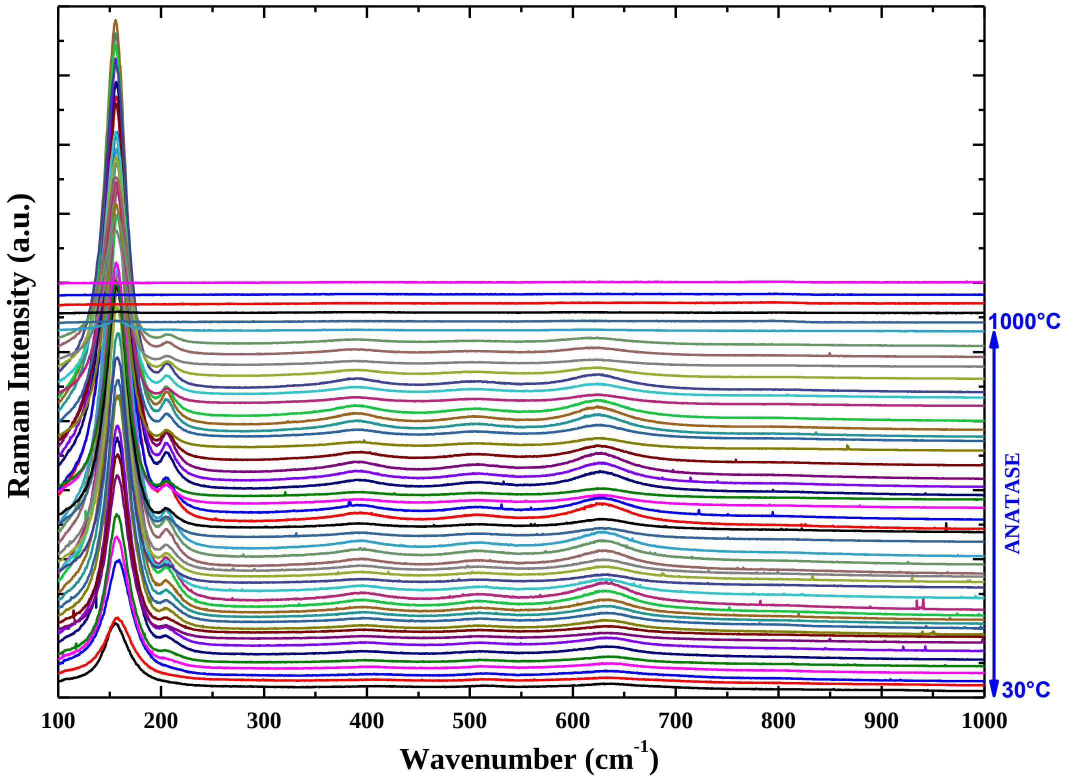

Phase stability frustration on ultra-nanosized anatase TiO_2

Snehangshu Patra,[1,2] Carine Davoisne,[1,2] Houssny Bouyanfif,[3] Dominique Foix,[2,4] Frédéric Sauvage[1,2]*

[1]Laboratoire de Réactivité et Chimie des Solides, Université de Picardie Jules Verne, CNRS UMR 7314, 33 rue Saint Leu, 80039 Amiens, France

[2]Réseau sur le Stockage Electrochimique de l'Energie (RS2E), FR CNRS 3459, France

[3]Laboratoire de Physique de la Matière Condensée, Université de Picardie Jules Verne, 33 rue Saint Leu, 80039 Amiens, France

[4]IPREM/ECP (CNRS UMR5254), University of Pau, Helioparc, 2 Av. Pierre Angot, 64053 Pau Cedex 9, France

Figure SI-3: Evolution of the particles morphology during first discharge of the 4-nm Li_xTiO_2 system.

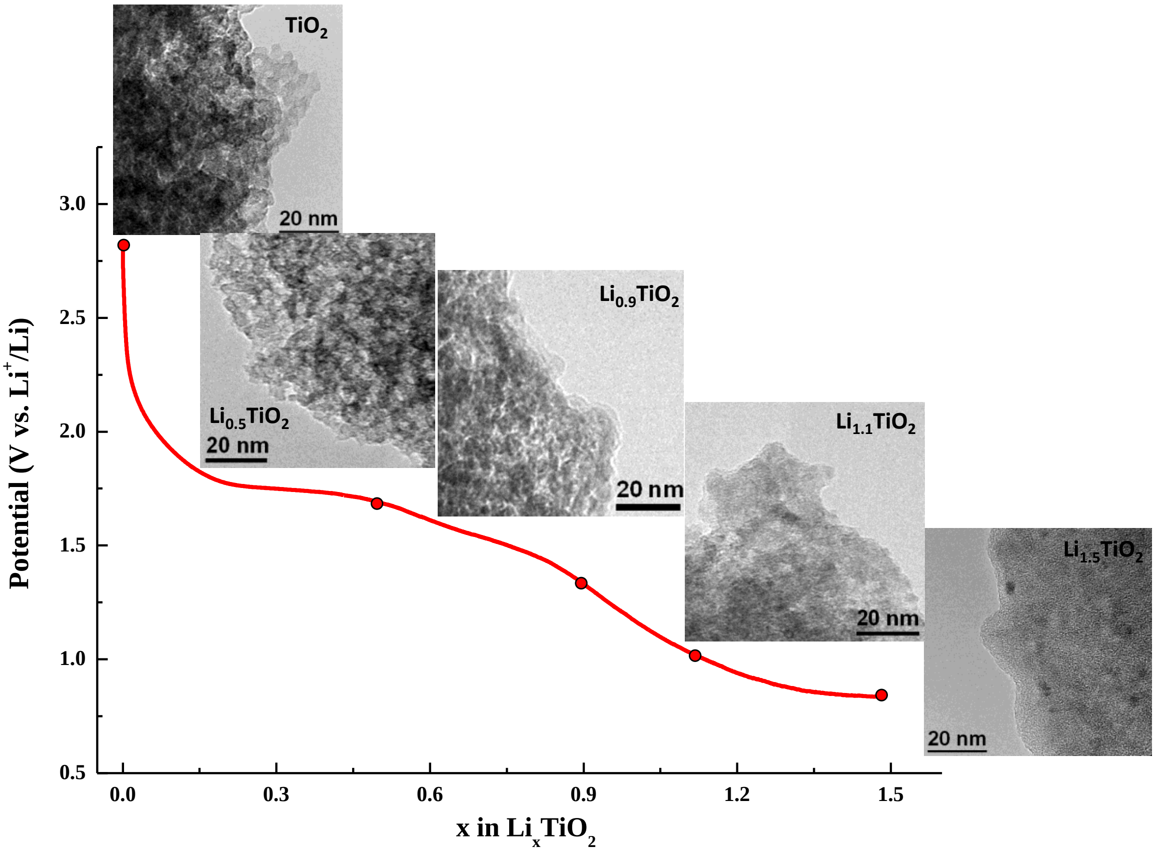

Supplement: Supplementary Information [file srep10928-s1.pdf]
